# Retrograde amnesia following electroconvulsive therapy for depression: propensity score analysis

**DOI:** 10.1192/bjo.2025.25

**Published:** 2025-04-04

**Authors:** Ana Jelovac, Sabine Landau, Gabriele Gusciute, Martha Noone, Keeva Kavanagh, Mary Carton, Cathal McCaffrey, Kelly McDonagh, Eimear Doody, Declan M. McLoughlin

**Affiliations:** Department of Psychiatry, Trinity College Dublin, St Patrick’s University Hospital, Dublin, Ireland; Department of Biostatistics and Health Informatics, King’s College London, London, UK; Trinity College Institute of Neuroscience, Trinity College Dublin, Dublin, Ireland

**Keywords:** Electroconvulsive therapy, autobiographical memory, retrograde amnesia, depressive disorders, bipolar type I or II disorders

## Abstract

Retrograde amnesia for autobiographical memories is a commonly self-reported cognitive side-effect of electroconvulsive therapy (ECT), but it is unclear to what extent objective performance differs between ECT-exposed and ECT-unexposed patients with depression. We investigated the association between exposure to brief-pulse (1.0 ms) bitemporal or high-dose right unilateral ECT and retrograde amnesia at short- and long-term follow-up, compared with inpatient controls with moderate-to-severe depression without lifetime exposure to ECT and receiving psychotropic pharmacotherapy and other aspects of routine inpatient care. In propensity score analyses, statistically significant reductions in autobiographical memory recall consistency were found in bitemporal and high-dose right unilateral ECT within days of an ECT course and 3 months following final ECT session. The reduction in autobiographical memory consistency was substantially more pronounced in bitemporal ECT. Retrograde amnesia for items recalled before ECT occurs with commonly utilised ECT techniques, and may be a persisting adverse cognitive effect of ECT.

Autobiographical retrograde amnesia is a commonly self-reported adverse cognitive effect of electroconvulsive therapy (ECT). Modifications in technique, including unilateral electrode placement and ultrabrief-pulse stimulus, attenuate retrograde amnesia.^1^ While the impact of ECT technique on autobiographical memory has been extensively documented, the extent to which autobiographical memory recall may be affected in ECT-exposed compared with ECT-unexposed patients with depression has received little attention. This is surprising, given the well-characterised autobiographical memory abnormalities in depression.^2^ Demonstrations of retrograde amnesia in ECT-exposed patients compared with non-depressed healthy controls are confounded by effects of mood disorder per se on autobiographical memory. To disentangle the effect of ECT on memory from that of mood disorder, the more appropriate control group is patients with depression of similar severity not receiving ECT. Early work examining retrograde amnesia as percentage loss of recall consistency of selected autobiographical memory items at post-ECT follow-ups, relative to content recalled at pre-ECT baseline, suggested that patients receiving bitemporal ECT exhibited worse recall consistency at the end of treatment than non-ECT-treated depressed controls.^3,4^ Two small contemporary studies compared ECT-exposed versus ECT-unexposed patients with depression. Patients receiving bifrontal ECT had reduced recall consistency at the end of treatment and 4-week follow-up compared with controls receiving isoflurane anaesthesia.^5^ One randomised trial compared autobiographical recall consistency following right unilateral ECT with a pharmacologically treated control group with bipolar depression; percentage consistency scores were 72.9 *v*. 80.8% at the end of treatment^6^ and 64.3 *v*. 72.3% at 6 months following ECT,^7^ respectively. Given the paucity of data quantifying retrograde amnesia in ECT patients compared with ECT-unexposed controls with depression, we aimed to examine the association between two commonly used forms of ECT and loss of autobiographical memory consistency.

## Method

Methods are detailed in the supplementary material (available at https://doi.org/10.1192/bjo.2025.25). ECT participants were recruited to the EFFECT-Dep Trial.^8^ Adult (age ≥18 years) in-patients were eligible if meeting Structured Clinical Interview for DSM-IV^9^ criteria for major depressive episode (unipolar or bipolar), and scoring ≥21 on the baseline 24-item Hamilton Depression Rating Scale (HAM-D). Exclusion criteria were (a) medical conditions rendering unfit for general anaesthesia; (b) ECT in previous 6 months; (c) history of schizophrenia, schizoaffective disorder or dementia; (d) substance use disorder in previous 6 months; and (e) involuntary status or inability to consent.

Control participants took part in MEM-Dep^10^ and AMBER-Dep^11^ prospective cohort studies, and were adult in-patients with major depressive episode meeting DSM-IV or ICD-10^12^ criteria, scoring ≥21 on HAM-D. Exclusion criteria were (a) history of ECT; (b) neurological or unstable medical condition; (c) active Axis I comorbidity; (d) substance use disorder in previous 6 months; and (e) involuntary status or inability to consent.

All groups received psychotropic medications and other aspects of inpatient care. This study received St Patrick’s Mental Health Services research ethics committee approval (Protocol no. 08/22). Informed consent requirements were waived for these secondary analyses of deidentified data.

Autobiographical memory was assessed using the Autobiographical Memory Interview–Short Form (AMI-SF)^13^ at pre-ECT baseline, end of treatment (within days of final ECT) and 3 months following final ECT. Controls were retested at analogous time intervals: 1–2 months following baseline assessment (coinciding with test–retest interval in ECT groups where patients received twice-weekly ECT) and 3 months following the second visit.

A propensity score stratification approach was used to control for measured confounding. Propensity scores were estimated for each comparison (right unilateral ECT versus control, bitemporal ECT versus control) at each time point (end of treatment and 3-month follow-up) using logistic regression models with seven putative confounders (age, gender, education, polarity, psychosis, baseline HAM-D and baseline AMI-SF score). Baseline covariate balance was considered adequate where (absolute) pooled within-strata standardised mean differences were <0.1. We estimated the average treatment effect on the treated (ATT) – here, the difference in average population recall percentages under ECT and control treatments for patients who later received ECT. Odds ratios arising from binomial models were converted to percentage differences using *g*-computation. Sensitivity analyses were conducted to evaluate the robustness of primary analyses to choice of analysis method and non-ignorable missingness.

## Results

Baseline characteristics of 210 included patients are provided in supplementary Table 1; supplementary Table 2 shows that baseline covariates were successfully balanced following propensity score stratification. In primary analyses (Table 1 and supplementary Fig. 1), AMI-SF percentage recall was significantly reduced in both ECT groups compared with ECT-unexposed depressed controls at the end of treatment and 3-month follow-up. Sensitivity analyses with alternative methods (Table 1) yielded similar ATT estimates, with reductions estimated at 7–10% for right unilateral ECT at sixfold seizure threshold and 18–21% for bitemporal ECT at 1.5-fold seizure threshold. There was a 24–25% loss of autobiographical recall consistency at both follow-ups in depressed controls (supplementary Table 3). Sensitivity analyses of non-ignorable missingness showed limited impact on ATT estimates (supplementary Table 4).

**Table 1:** Autobiographical Memory Interview–Short Form percentage recall differences between right unilateral ECT, bitemporal ECT and depressed control groups at end of treatment and 3-month follow-up

	Comparison			
	End of treatment		3-month follow-up	
	Right unilateral ECT versus control	Bitemporal ECT versus control	Right unilateral ECT versus control	Bitemporal ECT versus control
Primary analysis				
ATT from propensity score stratified binomial model^a^	−7.57% (−14.98 to −0.41%), *n* = 118	−18.68% (−23.90 to −13.21%), *n* = 121	−9.43% (−18.24 to −1.45%), *n* = 72	−21.04% (−27.51 to −9.75%), *n* = 72
Sensitivity analyses (alternative estimation approaches)				
ATT from IPTW binomial model^a^	−8.77% (−15.34 to −2.25%), *n* = 118	−17.90% (−23.03 to −12.15%), *n* = 121	−9.31% (−15.93 to −2.63%), *n* = 72	−20.78% (−28.86 to −12.86%), *n* = 72
ATT from naïve regression model with covariate adjustment	−8.48% (−14.18 to −2.78%), *n* = 133^b^	−18.33% (−24.05 to −12.61%), *n* = 133^b^	−7.71% (−14.63 to −0.79%), *n* = 93^b^	−18.93% (−26.84 to −11.02%), *n* = 85^b^

ATT, average treatment effect on the treated; ECT, electroconvulsive therapy; IPTW, inverse probability of treatment weighting.

a. Data presented as ATT (95% confidence interval); confidence intervals for ATT were generated by bootstrapping (2000 replications, percentile method).

b. Sample sizes are larger for naïve regression models compared with propensity score models, because the former do not use a region of common support.

## Discussion

We measured autobiographical memory consistency, an accepted measure of retrograde amnesia in the ECT field, in brief-pulse bitemporal ECT, high-dose right unilateral ECT and ECT-unexposed controls. Compared with a control group of in-patients with moderate-to-severe depression, both ECT techniques were associated with significantly increased loss of autobiographical memory consistency immediately following the course. The difference in autobiographical memory consistency between ECT groups and controls remained unattenuated in size and statistically significant at 3-month follow-up, implying that retrograde amnesia for AMI-SF items is a persisting side-effect of ECT. Differences between unilateral ECT and control groups at the end of treatment and at 3 months were similar to those from a randomised trial that found 7.9% difference at end of treatment and 8.0% at 6-month follow-up in a substantially different patient population (all bipolar and younger).^6,7^ An additional notable finding was that patients without exposure to ECT experienced substantial loss of autobiographical memory consistency over time. Consequently, when interpreting results of brain stimulation studies, the majority of which do not include a clinical control group, it is imperative not to misinterpret a within-group reduction in AMI-SF score as evidence of retrograde amnesia per se.

Brief-pulse bitemporal ECT, while falling out of favour at leading academic centres in North America, is in widespread use worldwide.^14^ The reluctance to switch to high-dose right unilateral ECT is difficult to justify given equivalent antidepressant efficacy and significantly reduced retrograde amnesia.^8,15^ Bitemporal ECT may result in more rapid reduction in depressive symptoms,^16^ which should be taken into consideration in scenarios where rapid response is required. However, for most ECT referrals for depression, this marginal benefit does not outweigh the markedly pronounced risk of retrograde amnesia. Ultrabrief-pulse right unilateral ECT, not examined in the present study, results in even less retrograde amnesia than brief-pulse right unilateral ECT, but this relative sparing of autobiographical memory may come at the expense of reduced efficacy.^17^ Risks and benefits of bitemporal versus alternative forms of ECT should be presented to patients during the informed consent process.

Limitations of this study include loss to 3-month follow-up and single-centre design. Future work is needed to address the limited evidence on impact of antidepressant medications on autobiographical memory. Our findings apply to the respective regions of common support used to achieve covariate balance. These regions excluded some ECT patients who had a negligible probability of being included in the control group. Our inferences regarding ECT are, therefore, applicable to less severely ill subpopulations. This is expected, because brief-pulse ECT occupies a special place in the treatment of the most severely ill psychiatric patients with no proven alternative treatment of comparable efficacy.

## Supporting information

Jelovac et al. supplementary material Jelovac et al. supplementary material

## Data Availability

An application to St Patrick’s Mental Health Services Research Ethics Committee with a study proposal is required for data sharing.

## Supplementary material

The supplementary material for this article can be found at https://doi.org/10.1192/bjo.2025.25
