# Supplementary material for: Retrograde amnesia following electroconvulsive therapy for depression: propensity score analysis
Source: BJPsych Open. 2025 Apr 4;11(3):e81. doi: 10.1192/bjo.2025.25 (PMC12052575; doi:10.1192/bjo.2025.25)
Supplement: Jelovac et al. supplementary material [file S2056472425000250sup001.docx]

**Supplementary Material**

**Retrograde amnesia following electroconvulsive therapy for depression: a propensity score analysis**

**Contents:**

**Data sources**

**ECT parameters**

**Autobiographical Memory Interview – Short Form (AMI-SF)**

**Supplementary statistical methods**

**Supplementary results**

**Supplementary references**

**Data sources**

Study participants were drawn from three previously reported studies.^1-3^ The EFFECT-Dep Trial^1^ was a pragmatic randomised, double-blind noninferiority trial of bitemporal versus high-dose right unilateral ECT. Control group patients with depression participated in two prospective cohort studies of autobiographical memory in depression, MEM-Dep^2^ and AMBER-Dep,^3^ the purpose of which was to validate the Autobiographical Memory Interview – Short Form (AMI-SF) and provide clinical control group data for this instrument.

**ECT parameters**

Patients were randomly assigned to twice-weekly brief-pulse ECT (1.0 ms pulse width; current amplitude 800 mA) at 1.5 × seizure threshold for bitemporal or 6 × seizure threshold for high-dose right unilateral (d’Elia) electrode placement. ECT was delivered using a MECTA spECTrum 5000M device (MECTA Corp, Tualatin, OR; maximum charge 1,200 mC). Seizure threshold was established using an empirical titration method as previously described.^1^ Stimulus dose was increased during the treatment course, as required. Methohexital (0.75–1 mg/kg) and succinylcholine (0.5–1.0 mg/kg) were used for anaesthesia and muscle relaxation. Duration of the ECT course and concomitant antidepressant therapy were at the discretion of referring psychiatrists.

**Autobiographical Memory Interview – Short Form (AMI-SF)**

The AMI-SF is a structured interview comprising 30 questions divided into six thematic sections assessing semantic and episodic autobiographical memory content regarding a family member, last major trip, last New Year’s Eve, last birthday, last employment, and last visit to a physician for a physical illness. At baseline, each answer may receive a score of 2 (specific answer provided) or 0 (no memory or vague answer), thus yielding a 0-60 range of possible total baseline scores. At follow-up visits, only specific answers scoring 2 at baseline are readministered. Follow-up answers may receive scores of 0, 1 or 2 depending on the degree of agreement with baseline answers. The AMI-SF yields a percentage score at post-ECT visits which is interpreted as consistency of recall at follow-up visits relative to answers provided at baseline. Higher percentage scores indicate better performance.

**Supplementary statistical methods**

The objectives of the statistical analyses were to assess differences in AMI-SF percentage recall between patients with depression treated with a form of ECT versus controls with depression unexposed to ECT at the end-of-treatment and 3-month follow-up after the final ECT session. As ECT treatment decisions are driven by patient characteristics which may also affect autobiographical memory recall, possible confounding needs to be addressed when comparing samples of ECT-exposed and unexposed patients. In addition, baseline variables might drive both loss to follow-up within ECT groups and AMI-SF recall and thus lead to missing data/selection bias. We carried out four separate analyses to estimate four contrasts: the recall difference between each form of ECT exposure relative to unexposed patients and for each of the two assessment timepoints (end-of-treatment and 3-month follow-up). Analyses samples were complete case samples and varied according to the form of ECT exposure under consideration as well as the assessment timepoint due to differential missingness in AMI-SF outcomes at end-of treatment and 3 months. The analyses are based on the assumption that all baseline variables acting as confounders or affecting sample selection have been observed. In other words, we assumed that there is no unobserved confounding and that the missing data generating mechanism is ignorable given baseline covariates.

Seven baseline variables were considered as potential confounders/sample selectors: age, gender, education level (≤12 years vs. >12 years), baseline depression severity (HAM-D score), polarity (unipolar vs. bipolar), presence of psychotic features (yes/no) during the current depressive episode and baseline AMI-SF score. We handled the multiple confounders/sample selectors by using propensity score methods, specifically propensity score stratification. In this context, the propensity score is a participant’s probability of ECT treatment assignment in the population of which the (selected) sample is representative. The propensity score is a balancing score, that is the analysis can be conditioned on the propensity score alone to remove biases due to observed baseline confounders/sample selectors. We estimated the propensity score from the relevant complete case sample and checked the balancing property empirically by calculating standardised differences (in units of within-group standard deviations) between control and ECT groups within propensity score strata. Standardised differences across strata of |d| < 0.1 were deemed adequate. More specifically, we (i) used logistic regression with potential baseline confounders as explanatory variables to predict propensity scores for the respective analysis sample, (ii) further restricted the sample to a region of common support, (iii) split this region into 5 equally spaced propensity score strata, and (iv) finally fitted an analysis model using these categorical strata to reflect the multiple confounders.

In terms of contrasts of interest, we specifically estimated the average treatment effects on the treated (ATT); here, the difference in average population recall percentages under ECT and control treatments for patients who later receive ECT treatment. The analysis model employed for this purpose modelled percentage recall by a binomial distribution for the number of items recalled out of the baseline AMI-SF count (denominator). We allowed for overdispersion. The explanatory variables in the model were treatment (ECT vs. no ECT), propensity score strata (5 levels) and terms modelling the interaction between group and strata. Since a binomial model naturally estimates an odds ratio, this was converted into an estimate of the percentage difference for the treated by using *g*-computation. Finally, 95% confidence intervals for ATT were generated by bootstrapping (2000 replications, percentile method).

Two sets of sensitivity analyses were performed. The first set was to check the sensitivity of our results to the choice of analysis method used to remove biases. We re-estimated average percentage differences using methods that made somewhat more restrictive assumptions. The first alternative approach was inverse probability of treatment weighting for the region of common support, which relies on the propensity score model being correct. The second alternative method was a naïve binomial regression model with baseline covariates included as additional explanatory variables and fitted to the whole sample. This approach relies on correctly modelling the relationship between any continuous covariate and proportion recall.

The second set of sensitivity analyses was carried out to assess the potential impact of non-ignorable dropout in an ECT group after the end-of-treatment. While most AMI-SF scores (response rate 197/210 = 93.81%) were available at the end-of-treatment assessment, there was considerable loss to follow-up at the 3-month timepoint (response rate 141/210 = 67.14%). We restricted our attention to those patients who provided AMI-SF scores at the end-of-treatment and considered scenarios whereby missing 3-month values in the depressed controls unexposed to ECT were occurring at random (i.e. not related to any variables), while missing values in the ECT-exposed groups were driven by the (later unobserved) AMI-SF values. Specifically, we imputed scenarios where those with worse recall were less likely to record a score. We imputed 8 analyses samples (4 samples for contrasting right unilateral ECT with controls and 4 for contrasting bitemporal ECT with controls) as follows:

- Within each group we calculated the mean percentage change in AMI-SF between end-of-treatment and 3-month assessment timepoints (relative to baseline) as well as the standard deviation of this change (SD percentage change).
- We always imputed any missing AMI-SF values in the control group by the score resulting for a patient whose percentage change between end-of-treatment and 3 months is equal to the mean percentage change for controls.
- For each ECT group we generated 4 different scenarios reflecting an increased worsening in recall at 3 months relative to end-of-treatment in those with missing AMI-SF: the percentage change in those with missing values is (A) the same as the mean percentage change in those who are observed; (B) is lower than this mean by 0.5 SD percentage change; (C) is lower by 1 SD; (D) is lower by 1.5 SD. We used these simulated change values to impute AMI-SF scores at 3 months.

These 8 completed analyses sets were then analysed using propensity score stratification and ATT was re-estimated each time. The change in an ATT estimate when moving from scenario (A) to scenario (D) reflects the impact of this increasingly informative missing data generating process. All data were analysed using Stata 18 (StataCorp, College Station, TX).

**Supplementary results**

*Clinical and demographic covariate balance before and after propensity score stratification*

Baseline AMI-SF scores were available for 80 depressed controls, 66 right unilateral ECT patients and 64 bitemporal ECT patients who completed baseline AMI-SF assessment. As expected, prior to propensity score stratification, the groups were not well balanced on covariates, with controls being younger, less severely depressed and far less likely to have a diagnosis of bipolar depression or psychotic depression (see Supplementary Table 1). Covariate balance following propensity score stratification is shown in Supplementary Table 2.

**Supplementary Table 1. Baseline demographic and clinical characteristics before propensity score stratification**

| **Variable** | **Total sample (n=210)** | **Depressed controls (n=80)** | **ECT type** | |
| --- | --- | --- | --- | --- |
|  |  |  | **Right unilateral (n=66)** | **Bitemporal (n=64)** |
| **Age, years, mean (SD)** | 54.83 (13.97) | 52.42 (11.90) | 56.09 (15.32) | 56.55 (14.69) |
| **Female, No. (%)** | 120 (57.14%) | 38 (47.50%) | 38 (57.58%) | 44 (68.75%) |
| **>12 years of education, No. (%)** | 71 (33.81%) | 33 (41.25%) | 23 (34.85%) | 15 (23.44%) |
| **Bipolar depression, No. (%)** | 36 (17.14%) | 6 (7.50%) | 16 (24.24%) | 14 (21.88%) |
| **Psychotic features, No. (%)** | 27 (12.16%) | 2 (2.50%) | 15 (22.73%) | 10 (15.63%) |
| **HAM-D score, mean (SD)** | 28.62 (5.88) | 26.65 (4.93) | 30.42 (6.13) | 29.23 (6.07) |
| **AMI-SF score, mean (SD)** | 46.76 (9.46) | 48.51 (8.13) | 46.88 (9.74) | 44.44 (10.35) |
| Abbreviations: AMI-SF: Autobiographical Memory Interview – Short Form; ECT: electroconvulsive therapy; HAM-D: 24-item Hamilton Depression Rating Scale | | | | |

**Supplementary Table 2. Summaries of baseline variables within regions of commons support and standardised mean differences within 5 equally spaced propensity score strata**

| **Variable** | **Comparison** | | | |
| --- | --- | --- | --- | --- |
|  | **End-of-treatment** | | **3-month follow-up** | |
|  | **Right unilateral ECT vs. control (n=118)** | **Bitemporal ECT vs. control (n=121)** | **Right unilateral ECT vs. control (n=72)** | **Bitemporal ECT vs. control (n=72)** |
| **Summary statistics for regions of common support (propensity score ranges from 0.05 to 0.90)** | | | | |
| **Age, mean (SD)** | 54.32 (12.98) | 53.43 (13.28) | 55.11 (12.45) | 54.79 (11.77) |
| **Female, No. (%)** | 64 (54.24%) | 67 (55.37%) | 34 (47.22%) | 37 (51.39%) |
| **>12 years of education, No. (%)** | 45 (38.14%) | 43 (35.54%) | 23 (31.94%) | 20 (27.78%) |
| **Bipolar depression, No. (%)** | 14 (11.86%) | 15 (12.40%) | 12 (16.67%) | 13 (18.06%) |
| **Psychotic features, No. (%)** | 6 (5.08%) | 4 (3.31%) | 3 (4.17%) | 3 (4.17%) |
| **HAM-D, mean (SD)** | 27.49 (5.06) | 27.01 (4.74) | 26.03 (4.08) | 25.68 (3.33) |
| **AMI-SF, mean (SD)** | 48.61 (7.98) | 47.60 (8.37) | 47.92 (8.63) | 47.58 (7.40) |
| **Standardised mean differences within propensity score strata** | | | | |
| **Propensity score** | 0.041 | 0.020 | 0.050 | 0.027 |
| **Age** | 0.026 | 0.020 | -0.070 | -0.013 |
| **Female** | 0.023 | 0.037 | -0.091 | 0.113 |
| **>12 years of education** | 0.018 | -0.019 | 0.148 | 0.001 |
| **Bipolar depression** | -0.003 | 0.047 | -0.045 | -0.005 |
| **Psychotic features** | -0.040 | -0.054 | 0.087 | -0.099 |
| **HAM-D** | 0.058 | 0.012 | 0.077 | 0.045 |
| **AMI-SF** | -0.002 | -0.002 | 0.066 | 0.021 |
| Abbreviations: AMI-SF: Autobiographical Memory Interview – Short Form; ECT: electroconvulsive therapy; HAM-D: 24-item Hamilton Depression Rating Scale | | | | |

At the end-of-treatment, percentage recall was measured for the majority of the sample (for 69/80 controls, 64/66 right unilateral ECT and 64/64 bitemporal ECT). At 3-month follow-up, only a subsample provided outcome measures (37/80 controls, 56/66 right unilateral ECT and 48/64 bitemporal ECT) due to loss to follow-up. Raw AMI-SF recall percentages are summarised in Supplementary Table 3 below.

**Supplementary Table 3. AMI-SF raw and percentage recall scores at end-of-treatment and 3-month follow-up**

| **Variable** | **Depressed controls** | **ECT type** | |
| --- | --- | --- | --- |
|  |  | **Right unilateral** | **Bitemporal** |
| **AMI-SF raw recall score at end-of-treatment, mean (SD)** | 37.23 (8.55)  n=69 | 31.08 (10.11)  n=64 | 25.13 (8.71)  n=64 |
| **AMI-SF percentage recall at end-of-treatment, mean (SD)** | 76.53% (11.77%), n=69 | 66.96% (16.29%), n=64 | 56.78% (17.30%), n=64 |
| **AMI-SF raw recall score at 3-month follow-up, mean (SD)** | 36.16 (9.00)  n=37 | 31.14 (9.55)  n=56 | 23.94 (8.07)  n=48 |
| **AMI-SF percentage recall at 3-month follow-up, mean (SD)** | 75.07% (13.23%), n=37 | 67.29% (14.19%), n=56 | 56.23% (18.13%), n=48 |
| Abbreviations: AMI-SF: Autobiographical Memory Interview – Short Form; ECT: electroconvulsive therapy | | | |

*Right unilateral ECT vs. no ECT at end-of-treatment*

As expected, all measured baseline covariates differed considerably between the right unilateral ECT and control groups for the subsample of patients who provided recall measures at the end-of-treatment (all standardised differences |d| > 0.1 for the covariates listed in Supplementary Table 1). The region of common support (chosen as the 0.05 to 0.90 propensity score range, n=118) excluded 15 patients, the majority of which were ECT treated patients who were deemed highly unlikely to be included in the control group of this analysis sample (e.g., due to psychotic depression or bipolar disorder diagnosis), compare top half of Supplementary Table 2 with Supplementary Table 1. After restricting the sample to the region of common support, all covariates were successfully balanced across groups within 5 equally spaced propensity score strata (all |SMD| < 0.1, see lower half of Supplementary Table 2).

The propensity score analysis estimated a statistically significant reduction of percentage recall in the right unilateral ECT group compared with controls (ATT = -7.57%, 95% percentile bootstrap interval from -14.98% to -0.41%). The sensitivity analyses gave similar results (naïve regression model: n=133, ATT = -8.48%, 95% CI from -14.18% to -2.78%; binomial model with inverse probability of treatment weighting: n=118, ATT = -8.77%, bootstrap percentile CI from -15.34% to -2.25%).

*Right unilateral ECT vs. no ECT at 3-month follow-up*

The comparison of patients receiving right unilateral ECT with controls at 3 months was based on a smaller sample (n=93). After restricting to the region of common support, n=72 contributed to the comparison (see top half of Supplementary Table 2). While the propensity scores clearly improved balance, one covariate did not meet the balance criterion (|SMD| = 0.15 for education, see lower half of Supplementary Table 2).

The estimated reduction in percentage recall after right unilateral ECT relative to controls was of similar size and again statistically significant at 3-month follow-up according to the propensity score stratification estimator of ATT = -9.43% (95% percentile bootstrap CI from -18.24% to -1.45%). The sensitivity analyses also estimated significant differences (naïve regression model: n=93, ATT = -7.71%, 95% CI from -14.63% to -0.79%; binomial model with inverse probability of treatment weighting: n=72, ATT = -9.31%, bootstrap percentile CI from -15.93% to -2.63%).

*Bitemporal ECT vs. no ECT at the end-of-treatment*

As expected, all measured baseline covariates differed between the bitemporal ECT group and the control group for the subsample of patients who provided recall measures at the end-of-treatment (all standardised differences |d| > 0.1 for covariates listed in Supplementary Table 1). The region of common support (chosen as the 0.05 to 0.90 propensity score range, n=121) excluded 12 patients which again were ECT treated patients who were deemed highly unlikely to be included in the control group of this analysis sample (see top half of Supplementary Table 2). After restricting the sample to the region of common support, all covariates were successfully balanced across groups within the propensity score strata (all |SMD| < 0.1, see lower half of Supplementary Table 2).

The propensity score analysis estimated a larger and statistically significant reduction of percentage recall in bitemporal ECT treated compared with controls (ATT = -18.68%, 95% percentile bootstrap CI from -23.90% to -13.21%). The sensitivity analyses gave similar results (naïve regression model: n=133, ATT = -18.33%, 95% CI from -24.05% to -12.61%; binomial model with inverse probability of treatment weighting: n=121, ATT = -17.90%, 95% bootstrap percentile CI from -23.03% to -12.15%).

*Bitemporal ECT vs. no ECT at 3-month follow-up*

The comparison of patients receiving bitemporal ECT with controls at 3 months was also based on a smaller sample (n=85). After restricting to the region of common support, n=72 contributed to the comparison. While the propensity scores clearly improved balance, one covariate again did not quite meet the balance criterion (|SMD| = 0.11 for gender; see lower half of Supplementary Table 2).

The estimated reduction in percentage recall after ECT treatment relative to controls at 3-month follow-up was of similar size as at the end-of-treatment and remained statistically significant according to the propensity stratification estimator of ATT = -21.04% (95% bootstrap percentile CI from -27.51% to -9.75%). The sensitivity analyses also indicated a significant reduction of a similar size (naïve regression model: n=85, ATT = -18.93%, 95% CI from -26.84% to -11.02%; binomial model with inverse probability of treatment weighting: n=72, ATT = -20.78%, 95% bootstrap percentile CI from -28.86% to -12.86%).

Results of the primary propensity score-stratified analyses are depicted in Supplementary Figure 1 below.

**Supplementary Figure 1. AMI-SF percentage recall differences between ECT and control groups at end-of-treatment and 3-month follow-up**

*Sensitivity analyses to assess the impact of non-ignorable AMI-SF missingness at 3 months*

The results of the second set of sensitivity analyses to assess the impact of non-ignorable AMI-SF missingness at 3 months are shown in Supplementary Table 4. This shows that under the “random dropout” scenario (A), ATT recall reduction estimates were somewhat lower than those generated by the primary analyses of the complete case samples. In addition, the ATT estimates from scenarios (B) – (D) show that if recall was increasingly worsened in those who do not provide data at 3 months, then the estimated reduction in recall in those receiving ECT relative to depressed controls is also increased (from 8.04% to 10.34% reduction under right unilateral ECT; from 20.12% to 26.03% reduction under bitemporal ECT). Taken together, these sensitivity analyses demonstrate that the substantive findings regarding the size of recall reductions after ECT relative to those experienced by depressed controls would be little impacted by such informative missingness.

**Supplementary Table 4. Sensitivity to non-ignorable missingness in those who dropped out from data collection after end-of-treatment within an ECT group**

|  | **3-month follow-up** | |
| --- | --- | --- |
|  | **Right unilateral ECT vs. control** | **Bitemporal ECT vs. control** |
| **Propensity score analysis (ignorable missingness, using complete case samples)** | | |
| ATT inference from propensity score stratified binomial model | -9.43%  (-18.24% to -1.45%), n=72 | -21.04%  (-27.51% to -9.75%), n=72 |
| **Sensitivity analyses (non-ignorable missingness, using imputed samples)** | | |
| ATT estimate if missing values in ECT group were imputed under scenario (A) | -8.04%  n=118 | -20.12%  n=121 |
| ATT estimate if missing values in ECT group were imputed under scenario (B) | -8.81%  n=118 | -22.03%  n=121 |
| ATT estimate if missing values in ECT group were imputed under scenario (C) | -9.57%  n=118 | -24.11%  n=121 |
| ATT estimate if missing values in ECT group were imputed under scenario (D) | -10.34%  n=118 | -26.03%  n=121 |
| Abbreviations: ATT: average treatment effect on the treated; ECT: electroconvulsive therapy  Estimated percentage recall differences between right unilateral ECT, bitemporal ECT and depressed control groups at 3-month follow-up if change in recall between end-of-treatment and 3-month follow-up in those who later dropped out is: (A) the same as the mean change in recall in those who are observed within the respective ECT group; (B) lower than this mean by 0.5 SD of change in recall; (C) lower by 1 SD; (D) lower by 1.5 SD. | | |

**Supplementary references**

1. Semkovska M, Landau S, Dunne R, Kolshus E, Kavanagh A, Jelovac A, et al. Bitemporal versus high-dose unilateral twice-weekly electroconvulsive therapy for depression (EFFECT-Dep): a pragmatic, randomized, non-inferiority trial. *Am J Psychiatry* 2016; **173(4)**: 408-417.
2. Semkovska M, Noone M, Carton M, McLoughlin DM. Measuring consistency of autobiographical memory recall in depression. *Psychiatry Res* 2012; **197(1-2)**: 41-48.
3. Whooley E, Gusciute G, Kavanagh K, McDonagh K, McCaffrey C, Doody E, et al. Reliable change indices and minimum detectable change for the Montreal Cognitive Assessment in electroconvulsive therapy for depression. *J ECT* [Epub ahead of print] Jul 5 2024. Available from: <https://doi:10.1097/YCT.0000000000001043>
